# Bis[*O*-propan-2-yl (4-eth­oxy­phen­yl)dithio­phospho­nato-κ^2^ *S*,*S*′]nickel(II)

**DOI:** 10.1107/S1600536812045114

**Published:** 2012-11-03

**Authors:** Shirveen Sewpersad, Werner E. Van Zyl

**Affiliations:** a School of Chemistry and Physics, University of KwaZulu-Natal, Westville Campus, Private Bag X54001, Durban 4000, South Africa

## Abstract

The title compound, [Ni(C_11_H_16_O_2_PS_2_)_2_], is a neutral four-coordinate mononuclear complex with a square-planar geometry. The complex lies on an inversion center. The metal atom is surrounded by two chelating isobidentate *O*-propan-2-yl (4-eth­oxy­phen­yl)dithio­phospho­nate ligands in a *trans* configuration binding through the S-donor atoms. The Ni—S bond lengths are 2.2328 (5) and 2.2369 (5) Å, an insignificant difference to be considered anisobidentate. The Ni⋯P separation is 2.8224 (5) Å and the S—P bond lengths are 2.0035 (7) and 2.0053 (7) Å. The S—Ni—S (chelating) and S—Ni—S (*trans*) bond angles are 88.321 (18) and 180°. The Ni—S—P bond angles are 83.26 (2) and 83.33 (2)°, indicating a very minor distortion from ideal square-planar geometry for the Ni atom. The P atom, however, is distorted quite significantly from an ideal tetra­hedral geometry, as reflected by the S—P—S and O—P—C bond angles of 101.93 (3) and 100.70 (7)°, respectively.

## Related literature

For information on dithio­phospho­nate compounds, see: Van Zyl & Fackler (2000 ▶); Van Zyl (2010 ▶). For examples of nickel(II) dithio­phospho­nate complexes, see: Liu *et al.* (2004 ▶); Gray *et al.* (2004 ▶); Aragoni *et al.* (2007 ▶); Arca *et al.* (1997 ▶); Malatesta & Pizzotti (1945 ▶); Hartung (1967 ▶).
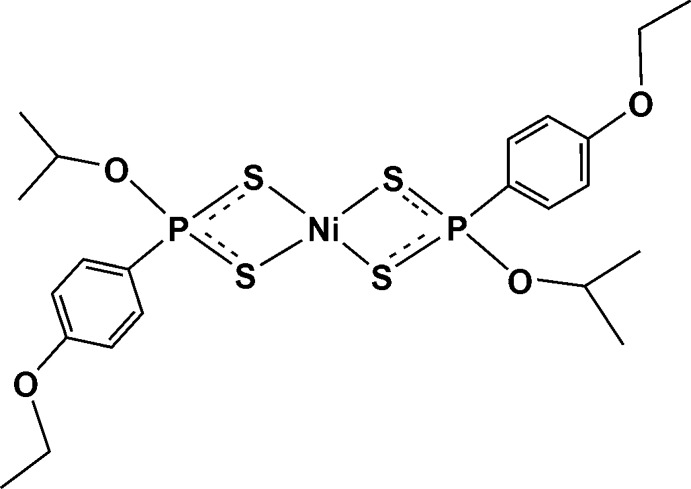


## Experimental

### 

#### Crystal data

[Ni(C_11_H_16_O_2_PS_2_)_2_]
*M* *_r_* = 609.37
Triclinic, 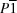

*a* = 7.8893 (6) Å
*b* = 8.4178 (7) Å
*c* = 11.4825 (10) Å
α = 109.530 (4)°
β = 101.959 (4)°
γ = 93.913 (5)°
*V* = 695.22 (10) Å^3^
*Z* = 1
Mo *K*α radiation
μ = 1.14 mm^−1^
*T* = 173 K
0.39 × 0.26 × 0.14 mm

#### Data collection

Nonius KappaCCD diffractometer
Absorption correction: multi-scan (*SADABS*; Sheldrick, 1996 ▶) *T*_min_ = 0.665, *T*_max_ = 0.857
14410 measured reflections
3062 independent reflections
2381 reflections with *I* > 2σ(*I*)
*R*_int_ = 0.035

#### Refinement

*R*[*F* ^2^ > 2σ(*F* ^2^)] = 0.030
*wR*(*F* ^2^) = 0.065
*S* = 1.02
3062 reflections
154 parameters
H-atom parameters constrained
Δρ_max_ = 0.27 e Å^−3^
Δρ_min_ = −0.31 e Å^−3^

### 

Data collection: *COLLECT* (Nonius, 2000 ▶); cell refinement: *DENZO-SMN* (Otwinowski & Minor, 1997 ▶); data reduction: *DENZO-SMN*; program(s) used to solve structure: *SHELXS97* (Sheldrick, 2008 ▶); program(s) used to refine structure: *SHELXL97* (Sheldrick, 2008 ▶); molecular graphics: *SHELXTL* (Sheldrick, 2008 ▶); software used to prepare material for publication: *SHELXL97*.

## Supplementary Material

Crystal structure: contains datablock(s) I, global. DOI: 10.1107/S1600536812045114/bh2460sup1.cif

Structure factors: contains datablock(s) I. DOI: 10.1107/S1600536812045114/bh2460Isup2.hkl

Additional supplementary materials:  crystallographic information; 3D view; checkCIF report

## Figures and Tables

**Fig. 1.**
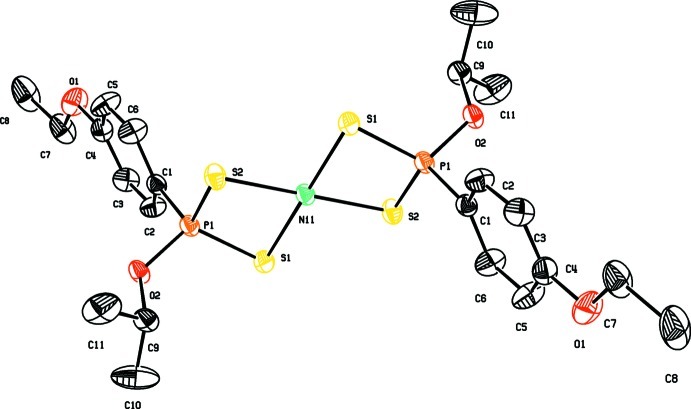
The molecular structure of the title complex, shown with 50% probability displacement ellipsoids.

## supplementary crystallographic information

### Comment

The phosphor-1,1,-dithiolate class of compounds is the heavier and softer congener of the more popular phosphonate derivatives. It contains the S_2_P functionality as a common feature and several sub-categories are known which include the dithiophosphato [S_2_P(O*R*')_2_]¯, (typically, *R*' = alkyl), dithiophosphinato [S_2_P*R*_2_]¯ (*R* = alkyl or aryl), and dithiophosphonato [S_2_P*R*(O*R*')]¯, (typically, *R* = aryl or ferrocenyl, *R*' = alkyl) monoanionic ligands. The latter may be described as a hybrid of the former two, and are also much less developed. Amongst all metals involved in the coordination chemistry of dithiophosphonato ligands, however, nickel(II) is by far the best represented (Aragoni *et al.*, 2007; Arca *et al.*, 1997; Liu *et al.*, 2004; Gray *et al.*, 2004), with the first example dating back to 1945 (Malatesta & Pizzotti, 1945) whilst the first X-ray structural report of a nickel(II) dithiophosphonate complex reported more than 2 decades later (Hartung, 1967). The complex in the present study was formed from the reaction between NiCl_2_.6H_2_0 and the ammonium salt of [S_2_P(O^i^Pr)(4-C_6_H_4_OEt)] (molar ratio 1:2) in an aqueous/methanolic solution, the NH_4_Cl by-product was dissolved and the precipitated product filtered off and washed with water. General and convenient methods to prepare dithiophosphonate salt derivatives have been reported (Van Zyl & Fackler, 2000; Van Zyl, 2010).

### Experimental

A colorless methanol (40 ml) solution of NH_4_[S_2_P(O^i^Pr)(4-C_6_H_4_OEt)] (982 mg, 3.347 mmol) was prepared. A second green solution of NiCl_2_.6H_2_0 (399 mg, 1.679 mmol) in deionized water (20 ml) was prepared, and added to the colorless solution with stirring over a period of 5 min. This resulted in a purple precipitate indicating the formation of the title complex. The precipitate was collected by vacuum filtration, washed with water (3 × 10 ml) and allowed to dry under vacuum for a period of 3 hrs, yielding a dry, free-flowing purple powder. Purple crystals suitable for X-ray analysis were grown by the slow diffusion of hexane into a dichloromethane solution of the title complex. Yield: 761 mg, 75%. *M*.p. 168–169°C.

^31^P NMR (CDCl_3_): δ (p.p.m.): 97.96. ^1^H NMR (CDCl_3_): δ (p.p.m.): 7.96 (2*H*, dd, *o*-ArH), 6.95 (2*H*, dd, *m*-ArH), 5.19 (1*H*, d quart, OCH), 4.06 (2*H*, quart, ArOCH_2_), 1.42 (3*H*, t, ArOCH_2_CH_3_), 1.38 (6*H*, d, CH_3_). ^13^C NMR (CDCl_3_): δ (p.p.m.): 162.22 (*p*-ArC), 131.71 (*m*-ArC), 128.04 (Ar—C_1_), 114.44 (*o*-ArC), 72.10 (CH), 63.76(ArOCH_2_), 24.30 (CH_3_), 14.67 (ArOCH_2_CH_3_).

### Refinement

All hydrogen atoms were found in the difference electron density maps and were placed in idealized positions and refined with geometrical constraints, with C—H bond lengths in the range 0.95-1.00 Å. The structure was refined to *R* factor of 0.0303.

### Crystal data

**Table:**

[Ni(C_11_H_16_O_2_PS_2_)_2_]	*Z* = 1
*M**_r_* = 609.37	*F*(000) = 318
Triclinic, *P*1	*D*_x_ = 1.455 Mg m^−^^3^
Hall symbol: -P 1	Melting point: 441 K
*a* = 7.8893 (6) Å	Mo *K*α radiation, λ = 0.71073 Å
*b* = 8.4178 (7) Å	Cell parameters from 14410 reflections
*c* = 11.4825 (10) Å	θ = 2.6–27.5°
α = 109.530 (4)°	µ = 1.14 mm^−^^1^
β = 101.959 (4)°	*T* = 173 K
γ = 93.913 (5)°	Block, purple
*V* = 695.22 (10) Å^3^	0.39 × 0.26 × 0.14 mm

### Data collection

**Table:**

Nonius KappaCCD diffractometer	3062 independent reflections
Radiation source: fine-focus sealed tube	2381 reflections with *I* > 2σ(*I*)
Graphite monochromator	*R*_int_ = 0.035
1.2° φ scans and ω scans	θ_max_ = 27.5°, θ_min_ = 2.6°
Absorption correction: multi-scan (*SADABS*; Sheldrick, 1996)	*h* = 0→10
*T*_min_ = 0.665, *T*_max_ = 0.857	*k* = −10→10
14410 measured reflections	*l* = −14→14

### Refinement

**Table:**

Refinement on *F*^2^	Primary atom site location: structure-invariant direct methods
Least-squares matrix: full	Secondary atom site location: difference Fourier map
*R*[*F*^2^ > 2σ(*F*^2^)] = 0.030	Hydrogen site location: inferred from neighbouring sites
*wR*(*F*^2^) = 0.065	H-atom parameters constrained
*S* = 1.02	*w* = 1/[σ^2^(*F*_o_^2^) + (0.028*P*)^2^ + 0.2462*P*] where *P* = (*F*_o_^2^ + 2*F*_c_^2^)/3
3062 reflections	(Δ/σ)_max_ < 0.001
154 parameters	Δρ_max_ = 0.27 e Å^−^^3^
0 restraints	Δρ_min_ = −0.31 e Å^−^^3^
0 constraints	

### Fractional atomic coordinates and isotropic or equivalent isotropic displacement parameters (Å^2^)

**Table:**

	*x*	*y*	*z*	*U*_iso_*/*U*_eq_	
Ni1	0.5000	0.5000	0.0000	0.02229 (10)	
S1	0.64099 (6)	0.75272 (6)	0.02356 (5)	0.02729 (13)	
S2	0.58144 (7)	0.38847 (6)	−0.18193 (5)	0.02842 (13)	
P1	0.64598 (6)	0.63370 (6)	−0.15849 (5)	0.02271 (12)	
O1	0.19743 (19)	0.8621 (2)	−0.53641 (14)	0.0416 (4)	
O2	0.82744 (15)	0.67472 (17)	−0.18857 (12)	0.0266 (3)	
C1	0.5013 (2)	0.7025 (2)	−0.26799 (17)	0.0231 (4)	
C2	0.4982 (3)	0.8752 (3)	−0.2422 (2)	0.0317 (5)	
H2	0.5664	0.9551	−0.1634	0.038*	
C3	0.3977 (3)	0.9335 (3)	−0.3289 (2)	0.0336 (5)	
H3	0.3976	1.0523	−0.3099	0.040*	
C4	0.2974 (2)	0.8179 (3)	−0.44330 (19)	0.0308 (5)	
C5	0.2947 (3)	0.6447 (3)	−0.4682 (2)	0.0405 (6)	
H5	0.2227	0.5647	−0.5455	0.049*	
C6	0.3960 (3)	0.5880 (3)	−0.3815 (2)	0.0360 (5)	
H6	0.3937	0.4690	−0.3996	0.043*	
C7	0.2176 (3)	1.0381 (3)	−0.5253 (2)	0.0463 (6)	
H7A	0.3430	1.0834	−0.5079	0.056*	
H7B	0.1696	1.1073	−0.4548	0.056*	
C8	0.1178 (3)	1.0441 (4)	−0.6510 (3)	0.0654 (8)	
H8A	0.1653	0.9734	−0.7200	0.098*	
H8B	0.1299	1.1621	−0.6482	0.098*	
H8C	−0.0063	1.0008	−0.6662	0.098*	
C9	0.9927 (2)	0.6387 (3)	−0.11992 (19)	0.0302 (5)	
H9	0.9693	0.6009	−0.0502	0.036*	
C10	1.1185 (3)	0.8017 (3)	−0.0629 (3)	0.0646 (8)	
H10A	1.1334	0.8447	−0.1302	0.097*	
H10B	1.2319	0.7811	−0.0211	0.097*	
H10C	1.0725	0.8863	0.0001	0.097*	
C11	1.0541 (3)	0.4983 (3)	−0.2128 (2)	0.0567 (7)	
H11A	0.9642	0.3971	−0.2481	0.085*	
H11B	1.1628	0.4711	−0.1695	0.085*	
H11C	1.0758	0.5343	−0.2819	0.085*	

### Atomic displacement parameters (Å^2^)

**Table:**

	*U*^11^	*U*^22^	*U*^33^	*U*^12^	*U*^13^	*U*^23^
Ni1	0.02098 (18)	0.0252 (2)	0.0246 (2)	0.00292 (14)	0.00633 (14)	0.01367 (16)
S1	0.0299 (3)	0.0276 (3)	0.0257 (3)	0.0000 (2)	0.0069 (2)	0.0119 (2)
S2	0.0343 (3)	0.0258 (3)	0.0305 (3)	0.0050 (2)	0.0128 (2)	0.0138 (2)
P1	0.0197 (2)	0.0268 (3)	0.0260 (3)	0.00378 (19)	0.0065 (2)	0.0145 (2)
O1	0.0440 (9)	0.0502 (10)	0.0364 (9)	0.0170 (7)	0.0024 (7)	0.0253 (8)
O2	0.0170 (6)	0.0397 (8)	0.0333 (8)	0.0077 (6)	0.0074 (6)	0.0248 (7)
C1	0.0184 (9)	0.0282 (11)	0.0257 (10)	0.0047 (8)	0.0065 (8)	0.0125 (9)
C2	0.0288 (11)	0.0299 (12)	0.0315 (12)	0.0026 (9)	−0.0009 (9)	0.0100 (10)
C3	0.0329 (11)	0.0284 (11)	0.0413 (13)	0.0073 (9)	0.0041 (10)	0.0170 (10)
C4	0.0259 (10)	0.0406 (13)	0.0323 (12)	0.0111 (9)	0.0076 (9)	0.0198 (10)
C5	0.0466 (14)	0.0352 (13)	0.0292 (12)	0.0069 (10)	−0.0062 (10)	0.0073 (10)
C6	0.0407 (12)	0.0283 (11)	0.0355 (12)	0.0090 (9)	0.0008 (10)	0.0112 (10)
C7	0.0392 (13)	0.0601 (16)	0.0630 (16)	0.0159 (11)	0.0154 (12)	0.0483 (14)
C8	0.0461 (15)	0.107 (2)	0.082 (2)	0.0262 (15)	0.0198 (14)	0.0779 (19)
C9	0.0194 (10)	0.0415 (12)	0.0363 (12)	0.0080 (9)	0.0029 (8)	0.0240 (10)
C10	0.0313 (13)	0.0465 (15)	0.097 (2)	0.0041 (11)	−0.0166 (14)	0.0217 (16)
C11	0.0352 (13)	0.0701 (18)	0.0548 (17)	0.0292 (12)	−0.0002 (12)	0.0123 (14)

### Geometric parameters (Å, º)

**Table:**

Ni1—S2	2.2328 (5)	C4—C5	1.387 (3)
Ni1—S2^i^	2.2328 (5)	C5—C6	1.378 (3)
Ni1—S1^i^	2.2369 (5)	C5—H5	0.9500
Ni1—S1	2.2369 (5)	C6—H6	0.9500
Ni1—P1	2.8224 (5)	C7—C8	1.513 (3)
Ni1—P1^i^	2.8224 (5)	C7—H7A	0.9900
S1—P1	2.0035 (7)	C7—H7B	0.9900
S2—P1	2.0053 (7)	C8—H8A	0.9800
P1—O2	1.5828 (12)	C8—H8B	0.9800
P1—C1	1.7894 (18)	C8—H8C	0.9800
O1—C4	1.364 (2)	C9—C11	1.489 (3)
O1—C7	1.438 (3)	C9—C10	1.497 (3)
O2—C9	1.484 (2)	C9—H9	1.0000
C1—C6	1.385 (3)	C10—H10A	0.9800
C1—C2	1.386 (3)	C10—H10B	0.9800
C2—C3	1.383 (3)	C10—H10C	0.9800
C2—H2	0.9500	C11—H11A	0.9800
C3—C4	1.381 (3)	C11—H11B	0.9800
C3—H3	0.9500	C11—H11C	0.9800
			
S2—Ni1—S2^i^	180.0	O1—C4—C5	116.20 (19)
S2—Ni1—S1^i^	91.679 (18)	C3—C4—C5	119.59 (18)
S2^i^—Ni1—S1^i^	88.321 (18)	C6—C5—C4	120.3 (2)
S2—Ni1—S1	88.321 (18)	C6—C5—H5	119.8
S2^i^—Ni1—S1	91.679 (18)	C4—C5—H5	119.8
S1^i^—Ni1—S1	180.0	C5—C6—C1	120.7 (2)
S2—Ni1—P1	44.885 (16)	C5—C6—H6	119.6
S2^i^—Ni1—P1	135.115 (17)	C1—C6—H6	119.6
S1^i^—Ni1—P1	135.174 (16)	O1—C7—C8	106.7 (2)
S1—Ni1—P1	44.827 (16)	O1—C7—H7A	110.4
S2—Ni1—P1^i^	135.115 (16)	C8—C7—H7A	110.4
S2^i^—Ni1—P1^i^	44.885 (16)	O1—C7—H7B	110.4
S1^i^—Ni1—P1^i^	44.827 (16)	C8—C7—H7B	110.4
S1—Ni1—P1^i^	135.173 (16)	H7A—C7—H7B	108.6
P1—Ni1—P1^i^	180.0	C7—C8—H8A	109.5
P1—S1—Ni1	83.26 (2)	C7—C8—H8B	109.5
P1—S2—Ni1	83.33 (2)	H8A—C8—H8B	109.5
O2—P1—C1	100.70 (7)	C7—C8—H8C	109.5
O2—P1—S1	113.76 (6)	H8A—C8—H8C	109.5
C1—P1—S1	113.38 (7)	H8B—C8—H8C	109.5
O2—P1—S2	114.16 (6)	O2—C9—C11	107.84 (16)
C1—P1—S2	113.47 (7)	O2—C9—C10	107.22 (17)
S1—P1—S2	101.93 (3)	C11—C9—C10	113.9 (2)
O2—P1—Ni1	141.40 (5)	O2—C9—H9	109.3
C1—P1—Ni1	117.90 (6)	C11—C9—H9	109.3
S1—P1—Ni1	51.913 (17)	C10—C9—H9	109.3
S2—P1—Ni1	51.790 (18)	C9—C10—H10A	109.5
C4—O1—C7	118.46 (17)	C9—C10—H10B	109.5
C9—O2—P1	121.36 (11)	H10A—C10—H10B	109.5
C6—C1—C2	118.40 (18)	C9—C10—H10C	109.5
C6—C1—P1	121.83 (15)	H10A—C10—H10C	109.5
C2—C1—P1	119.72 (15)	H10B—C10—H10C	109.5
C3—C2—C1	121.31 (19)	C9—C11—H11A	109.5
C3—C2—H2	119.3	C9—C11—H11B	109.5
C1—C2—H2	119.3	H11A—C11—H11B	109.5
C4—C3—C2	119.60 (19)	C9—C11—H11C	109.5
C4—C3—H3	120.2	H11A—C11—H11C	109.5
C2—C3—H3	120.2	H11B—C11—H11C	109.5
O1—C4—C3	124.21 (19)		
			
S2—Ni1—S1—P1	12.58 (2)	S1—P1—O2—C9	58.46 (15)
S2^i^—Ni1—S1—P1	−167.42 (2)	S2—P1—O2—C9	−57.97 (15)
P1^i^—Ni1—S1—P1	180.0	Ni1—P1—O2—C9	0.38 (19)
S1^i^—Ni1—S2—P1	167.43 (2)	O2—P1—C1—C6	101.89 (17)
S1—Ni1—S2—P1	−12.57 (2)	S1—P1—C1—C6	−136.21 (15)
P1^i^—Ni1—S2—P1	180.0	S2—P1—C1—C6	−20.53 (18)
Ni1—S1—P1—O2	−137.71 (6)	Ni1—P1—C1—C6	−78.31 (17)
Ni1—S1—P1—C1	107.99 (7)	O2—P1—C1—C2	−75.50 (16)
Ni1—S1—P1—S2	−14.35 (3)	S1—P1—C1—C2	46.41 (17)
Ni1—S2—P1—O2	137.47 (6)	S2—P1—C1—C2	162.08 (14)
Ni1—S2—P1—C1	−107.90 (7)	Ni1—P1—C1—C2	104.30 (15)
Ni1—S2—P1—S1	14.37 (3)	C6—C1—C2—C3	−2.3 (3)
S2—Ni1—P1—O2	−81.30 (9)	P1—C1—C2—C3	175.20 (15)
S2^i^—Ni1—P1—O2	98.70 (9)	C1—C2—C3—C4	0.4 (3)
S1^i^—Ni1—P1—O2	−99.27 (9)	C7—O1—C4—C3	10.4 (3)
S1—Ni1—P1—O2	80.73 (9)	C7—O1—C4—C5	−169.75 (19)
S2—Ni1—P1—C1	99.02 (8)	C2—C3—C4—O1	−178.27 (18)
S2^i^—Ni1—P1—C1	−80.98 (8)	C2—C3—C4—C5	1.9 (3)
S1^i^—Ni1—P1—C1	81.05 (8)	O1—C4—C5—C6	177.9 (2)
S1—Ni1—P1—C1	−98.95 (8)	C3—C4—C5—C6	−2.2 (3)
S2—Ni1—P1—S1	−162.03 (3)	C4—C5—C6—C1	0.3 (3)
S2^i^—Ni1—P1—S1	17.97 (3)	C2—C1—C6—C5	1.9 (3)
S1^i^—Ni1—P1—S1	180.000 (1)	P1—C1—C6—C5	−175.49 (17)
S2^i^—Ni1—P1—S2	180.0	C4—O1—C7—C8	169.83 (18)
S1^i^—Ni1—P1—S2	−17.97 (3)	P1—O2—C9—C11	112.06 (18)
S1—Ni1—P1—S2	162.03 (3)	P1—O2—C9—C10	−124.87 (18)
C1—P1—O2—C9	−179.91 (14)		

Symmetry code: (i) −*x*+1, −*y*+1, −*z*.
